# Systemic Delivery of Tyrosine-Mutant AAV Vectors Results in Robust Transduction of Neurons in Adult Mice

**DOI:** 10.1155/2013/974819

**Published:** 2013-05-20

**Authors:** Asako Iida, Naomi Takino, Hitomi Miyauchi, Kuniko Shimazaki, Shin-ichi Muramatsu

**Affiliations:** ^1^Division of Neurology, Department of Medicine, Jichi Medical University, 3311-1 Yakushiji, Shimotsuke, Tochigi 329-0498, Japan; ^2^Division of Neurosurgery, Jichi Medical University, 3311-1 Yakushiji, Shimotsuke, Tochigi 329-0498, Japan

## Abstract

Recombinant adeno-associated virus (AAV) vectors are powerful tools for both basic neuroscience experiments and clinical gene therapies for neurological diseases. Intravascularly administered self-complementary AAV9 vectors can cross the blood-brain barrier. However, AAV9 vectors are of limited usefulness because they mainly transduce astrocytes in adult animal brains and have restrictions on foreign DNA package sizes. In this study, we show that intracardiac injections of tyrosine-mutant pseudotype AAV9/3 vectors resulted in extensive and widespread transgene expression in the brains and spinal cords of adult mice. Furthermore, the usage of neuron-specific promoters achieved selective transduction of neurons. These results suggest that tyrosine-mutant AAV9/3 vectors may be effective vehicles for delivery of therapeutic genes, including miRNAs, into the brain and for treating diseases that affect broad areas of the central nervous system.

## 1. Introduction

Various gene delivery carriers have been tested in preclinical gene therapies for diseases that affect the central nervous system (CNS). Based on the results of these studies, adeno-associated virus (AAV) derived vectors are the most suitable for clinical applications because they are both efficacious and safe [1–3]. Infusions of recombinant AAV vectors via stereotaxic surgeries into target brain areas result in continuous and long-term expression of transgenes [4, 5]. Several phase I/II gene therapy trials for Parkinson's disease, in which therapeutic genes were introduced into the putamen or subthalamic nucleus, demonstrate encouraging clinical benefits [5–8]. However, for diseases that affect large areas of the CNS, such as Alzheimer's disease, lipid storage diseases, and multiple sclerosis, local injections of the vectors yield suboptimal results. Vector deliveries through the vasculature system may achieve more widespread transductions of the viruses. 

Vectors derived from AAV type 9 (AAV9) have recently become popular because they cross the blood-brain barrier (BBB) or the blood-cerebrospinal fluid barrier [9–14]. However, while intravenous injections of AAV9 vectors achieved efficient transduction of spinal motor neurons in fetal, neonate, and adult mice, as well as in adult cats and pigs [9–13, 15, 16], most of the transduced cells were astrocytes in adult mice and nonhuman primates [9, 14, 16]. Thus, extensive gene delivery to neurons in the adult CNS remains challenging. 

Most previous reports about systemic delivery of AAV9 vectors to the CNS used self-complementary AAV (scAAV) vectors with two complementary copies of a transgene that were inserted at the expense of maintaining small packaging sizes (less than 2.2 kb) [17]. scAAV vectors are 20- to 100-fold more efficient than conventional single strand AAV vectors [18], but packaging constraints set strict size limits on the genes that can be delivered. Furthermore, AAV9 vectors with a cytomegalovirus (CMV) promoter can transduce antigen-presenting cells in the brain and provoke an adaptive immune response that results in significant brain pathology [19]. This immune response presents an additional obstacle for the usage of AAV9 vectors in the CNS. Usage of neuron-specific promoters may circumvent this strong immune reaction. However, many cell-type specific promoters drive relatively weak gene expression [20]. Therefore, we devised a novel approach in order to improve transgene expression. Specifically, we eliminated two surface-exposed tyrosine residues from the capsid protein of AAV9. Substituting highly conserved surface-exposed capsid tyrosine residues for phenylalanine residues results in increased infectivities for several AAV vectors [21–27]. Our results in this study demonstrate that tyrosine-mutant pseudotype AAV9/3 vectors with neuron-specific promoters can achieve extensive gene expression in neurons.

## 2. Materials and Methods

### 2.1. Generation of Pseudotype AAV9/3 Vectors

The AAV vector plasmids contained an expression cassette consisting of a promoter, which was followed by cDNA encoding either green fluorescent protein (GFP) or the microRNA (miRNA) sequence for human aromatic l-amino acid decarboxylase (AADC) and then a woodchuck hepatitis virus posttranscriptional regulatory element. The expression cassette was located between the inverted terminal repeats of the AAV type 3 (AAV3) genome. Three distinct promoters were used: a human cytomegalovirus immediate-early enhancer and chicken *β*-actin (CAG) promoter, the neuron-specific synapsin I promoter (Gene Bank, M55300.1) [28], or the Purkinje cell-specific L7 promoter (Gene Bank, S40022.1) [29]. A double strand DNA sequence encoding the miRNA for human AADC was synthesised with the following sequence: 5′-TGCTGAATTCAGGACAGATAAAGGCAGTTTTGGCCACTGACTGACTGCCTTTATGTCCTGAATT-3′. The AAV9 *vp* cDNA was synthesised as previously described [30], except that substitutions of thymidine for adenine were inserted at positions 1337 and 2192. These substitutions introduced amino acid changes from tyrosine to phenylalanine at positions 446 and 731. The recombinant AAV vectors were produced by transient transfection of HEK293 cells, as previously described [31]. The cells were transfected with the vector plasmid, the AAV3 *rep* and AAV9 *vp* expression plasmids, and the adenoviral helper plasmid pHelper (Invitrogen). The recombinant viruses were purified by isolation from two sequential continuous CsCl gradients. Finally, the viral titers were determined by qRT-PCR.

### 2.2. Intracardiac Vector Injections in Adult Mice

All animal experiments were performed in compliance with institutional guidelines. Twenty-four male, C57BL/6, 9-10-week-old mice were included in this study. The mice were housed in plastic cages, had ad lib access to food and water, and were maintained on a 12/12 h light-dark cycle. For injections, AAV vectors were diluted in phosphate-buffered saline (PBS) to 1.2 × 10^11^– 8.5 × 10^12^ vectors genome/100 *μ*L. Mice were anaesthetised with pentobarbital (50 mg/kg, ip), and then 100 *μ*L of the diluted AAV vectors was intracardially injected with a 0.5 mL syringe equipped with a 29-gauge needle. 

### 2.3. Immunohistochemistry

Four to eight weeks after injections of vectors, the mice were anaesthetised with pentobarbital and perfused with ice-cold 4% paraformaldehyde in PBS. The brains, hearts, livers, and kidneys were dissected, postfixed in the same solution, cryoprotected with 30% sucrose in PBS for 48 h, and then frozen. Coronal sections (thickness of 40 *μ*m) were cut on a microtome with a freezing unit, collected in PBS (pH 7.4), and divided into series. Tissue sections were incubated overnight with primary antibodies at 4°C. The primary antibodies used, their sources, and the dilutions used for immunohistochemistry were GFP (chicken, Abcam, 1 : 1,000–1 : 10,000; or rabbit, Abcam, 1 : 1000); tyrosine hydroxylase (TH) (mouse, DiaSorin, 1 : 800); AADC (rabbit, 1 : 5000; provided by Nagatsu I., Fujita Medical University); the neuronal marker NeuN (mouse, Millipore, 1 : 100); the astrocyte marker glial fibrillary acidic protein (GFAP) (rabbit, Covance, 1 : 1000); the Choline Acetyltransferase (ChAT) (mouse, CHEMICON, 1 : 200); and the Purkinje cell marker calbindin (mouse, SIGMA, 1 : 1000). The secondary antibodies used, their sources, and the dilutions used to detect the primary antibodies were Alexa Fluor 488 goat anti-chicken IgG (1 : 1000; Invitrogen); Alexa Fluor 594 goat anti-mouse IgG (1 : 1000; Invitrogen); Alexa Fluor 405 goat anti-mouse IgG (1 : 200; Invitrogen); and Alexa Fluor 594 goat anti-rabbit IgG (1 : 1000; Invitrogen). Immunofluorescent signals were assessed with a confocal laser scanning microscope (FV10i; Olympus, Tokyo). 

### 2.4. Quantification of Gene Expression

The numbers of GFP/NeuN double-positive cells in the brain were counted using a stereological method. One in every five coronal sections covering either the frontal cortex, hippocampus, or amygdala of vector-injected mice was processed. Cell counting was performed in total on eight sections of each region of interest. Magnified images were taken using a 10 × objective lens (NA 0.5) at multiple different focal planes to visualize all cells in the thickness.

## 3. Results

### 3.1. Global Brain Transduction Was Achieved with Tyrosine-Mutant AAV9/3 Vectors

**Figure 1:**
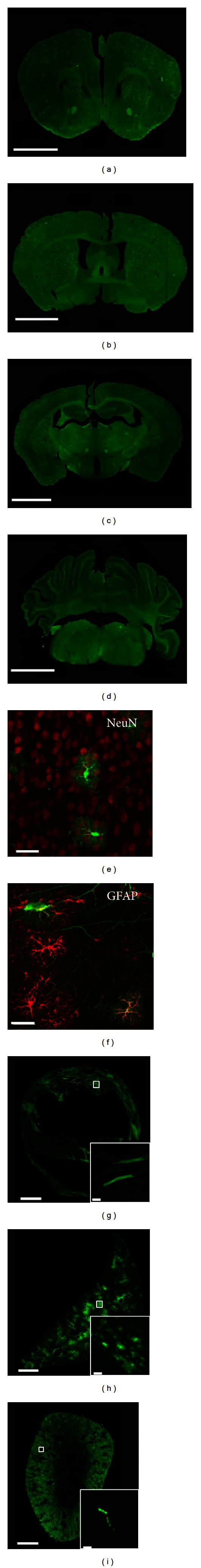
Widespread preferential glial transduction of mouse brains after intracardiac injections of tyrosine-mutant AAV9/3 vectors containing a ubiquitous promoter. The tyrosine-mutant AAV9/3 vector expressed green fluorescent protein (GFP) under control of the cytomegalovirus immediate-early enhancer and chicken *β*-actin (CAG) promoter. ((a)–(d)) Representative images of coronal sections stained with an anti-GFP antibody following virus administration. Section locations relative to the Bregma: (a) +1.34 mm; (b) +0.14 mm; (c) –1.70 mm; and (d) –6.24 mm. ((e), (f)) Merged images showing results of double immunostaining experiments. Most transduced cells showed glial morphologies and were immunoreactive for glial fibrillary acidic protein (GFAP) but not NeuN. (e) GFP (green), NeuN (red); and (f) GFP (green), GFAP (red). Analysis of GFP expression in peripheral organs revealed robust transduction of the heart, liver, and kidney (g–i). Scale bars: ((a)–(d)) 2 mm; ((e), (f), insets of (g)–(i)) 30 *μ*m; ((g)–(i)) 1 mm.

We generated pseudotype tyrosine-mutant AAV9/3 vectors, administered the vectors to adult mice with intracardiac injections, and then evaluated brain transduction. Mutant AAV9 capsid proteins containing two residues, where phenylalanine was substituted for tyrosine (Y446F and Y731F), encapsulated the vectors. The vectors were also engineered to express GFP under control of the CAG promoter (yfAAV9/3-CAG-GFP). Four weeks after administration (1.2 × 10^11^ vectors genome/mouse), widespread and extensive brain transductions were observed (Figures 1(a)–1(d)). Immunohistochemistry experiments demonstrated that most GFP-immunoreactive (GFP-IR) cells had glial cell morphologies and expressed the glial cell marker glial fibrillary acidic protein (GFAP) (Figure 1(f)). Robust GFP expression was observed in peripheral organs including the heart, liver, and kidney (Figures 1(g)–1(i)).

### 3.2. Neuronal Transduction Was Achieved with Specific Promoters

**Figure 2:**
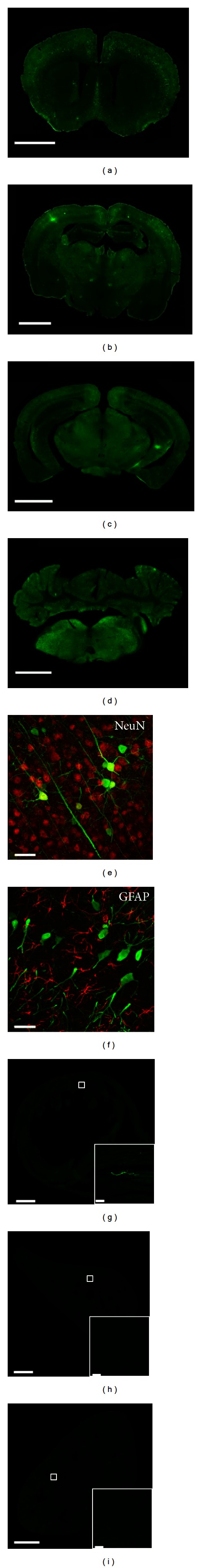
Efficient transduction of neurons with intracardiac injections of the tyrosine-mutant AAV9/3 vectors containing a neuron-specific promoter. Extensive transduction of neurons was achieved throughout the mouse brain by injections of tyrosine-mutant AAV9/3 vectors that expressed GFP under control of the synapsin I promoter. Representative images of coronal sections stained with an anti-GFP antibody following virus administration. Section locations relative to the Bregma: (a) +0.74 mm; (b) –1.82 mm; (c) –2.92 mm; and (d) –6.12 mm. ((e), (f)) Merged images showing results of double immunostaining experiments. Nearly all transduced cells were immunoreactive for NeuN but not GFAP. (e) GFP (green), NeuN (red); and (f) GFP (green), GFAP (red). Analysis of peripheral organs in yfAAV9/3-SynI-GFP vector-injected mice revealed that GFP expression was not observed in heart muscle, kidney cells, or hepatocytes ((g)–(i)) and was restricted to peripheral nerve cells (inset in (g)). Scale bars: ((a)–(d)) 2 mm; ((e), (f), and inset of (g)–(i)) 30 *μ*m; ((g)–(i)) 1 mm.

**Figure 3:**
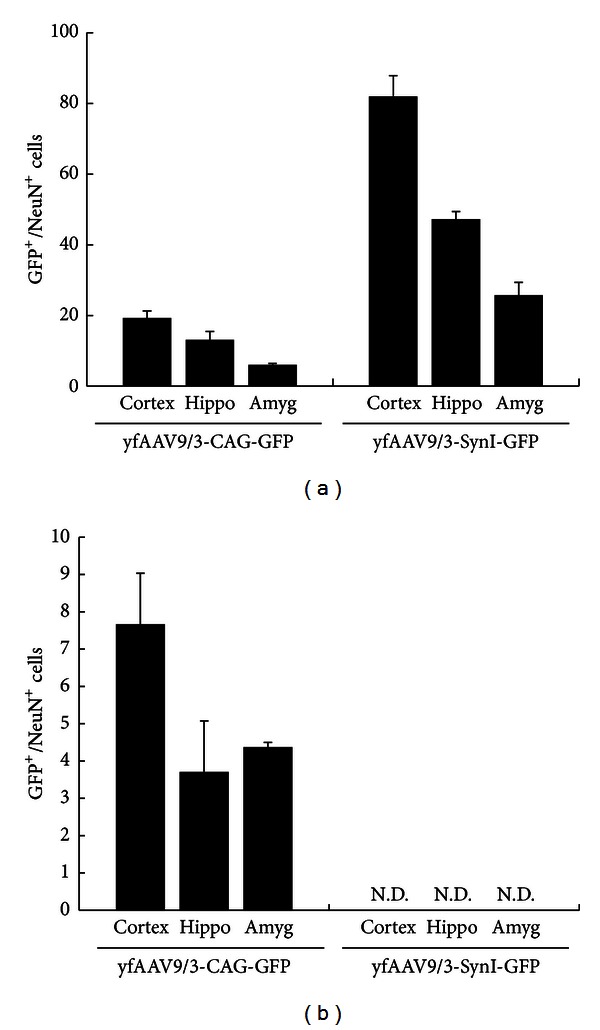
Quantification of neuronal transduction. The numbers of GFP^+^/NeuN^+^ neuronal or GFP^+^/NeuN^−^ nonneuronal cells in yfAAV-CAG-GFP-injected and yfAAV-SynI-GFP-injected mice (*n* = 6) were compared. The yfAAV9/3-SynI-GFP vector transduced approximately four times more NeuN-positive neurons than the yfAAV9/3-CAG-GFP vector in the frontal cortex, hippocampus, and amygdala. The volume of each region of interest was 0.04 × 1 × 1 mm^3^. A total of eight sections of each region of interest were analyzed. N.D., not detected; Hippo, hippocampus; Amyg, amygdala.

**Figure 4:**
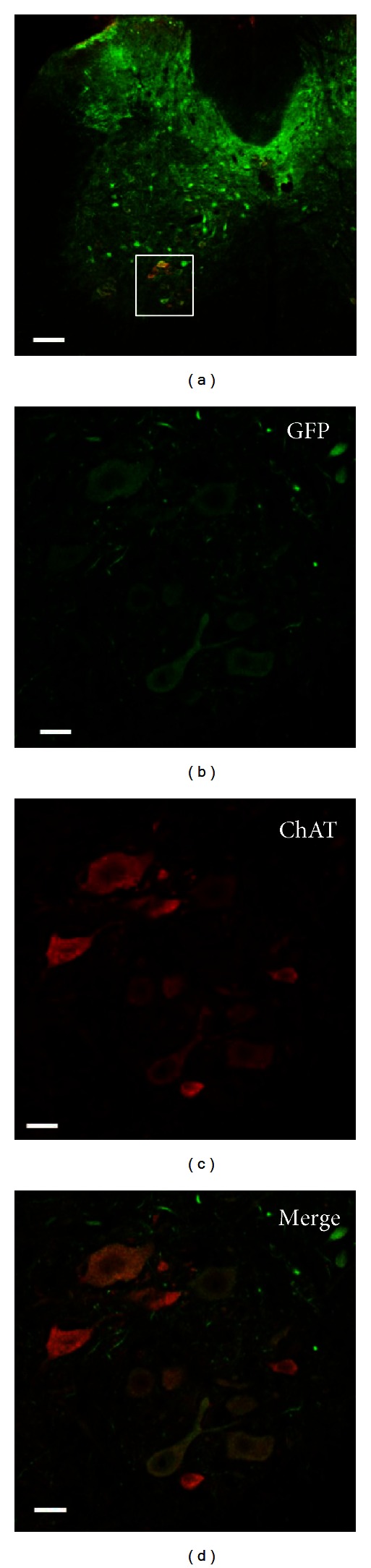
Transduction of spinal cord motor neurons. Immunohistochemical detection of GFP and choline acetyl transferase (ChAT), a motor neuron marker, in mice spinal cords following intracardiac injections of tyrosine-mutant AAV9/3 vectors that expressed GFP under control of the synapsin I promoter. (a) An axial section demonstrating colabeling of GFP (green) and ChAT (red) in the large ventral horn neurons of the spinal cord. ((b)–(d)) High magnification images of the square area outlined in (a). (b) GFP-positive cells (green); (c) ChAT-positive cells (red); and (d) the merged image of ((b) and (c)). Scale bars: (a) 100 *μ*m; ((b)–(d)) 20 *μ*m.

We next made AAV9/3 vectors that expressed GFP under control of the neuron-specific synapsin I promoter and were encapsulated with double tyrosine-mutant capsid proteins (yfAAV9/3-SynI-GFP). Six weeks after intracardiac infusions of the vectors into adult mice, robust transductions were observed throughout the brains (Figures 2(a)–2(d)). Double immunofluorescence stainings demonstrated that nearly all GFP-IR cells expressed the neuronal marker NeuN (NeuN-IR) (Figure 2(e)). When the yfAAV9/3-SynI-GFP vector was employed, GFP expression was not observed in peripheral organs but was observed in peripheral neurons (Figures 2(g)–2(i)). The number of GFP/NeuN double-positive cells in the frontal cortex, hippocampus, and amygdala of yfAAV9/3-SynI-GFP vector-injected mice was counted and compared with that in yfAAV9/3-CAG-GFP vector-injected mice. The yfAAV9/3-SynI-GFP vector transduced approximately four times more neurons than the yfAAV9/3-CAG-GFP vector without transducing NeuN-negative nonneuronal cells (Figure 3). Furthermore, immunofluorescent examinations of spinal cord sections revealed GFP-IR motor neurons that also expressed the motor neuron marker choline acetyl transferase (ChAT) in the ventral horns (Figure 4). 

**Figure 5:**
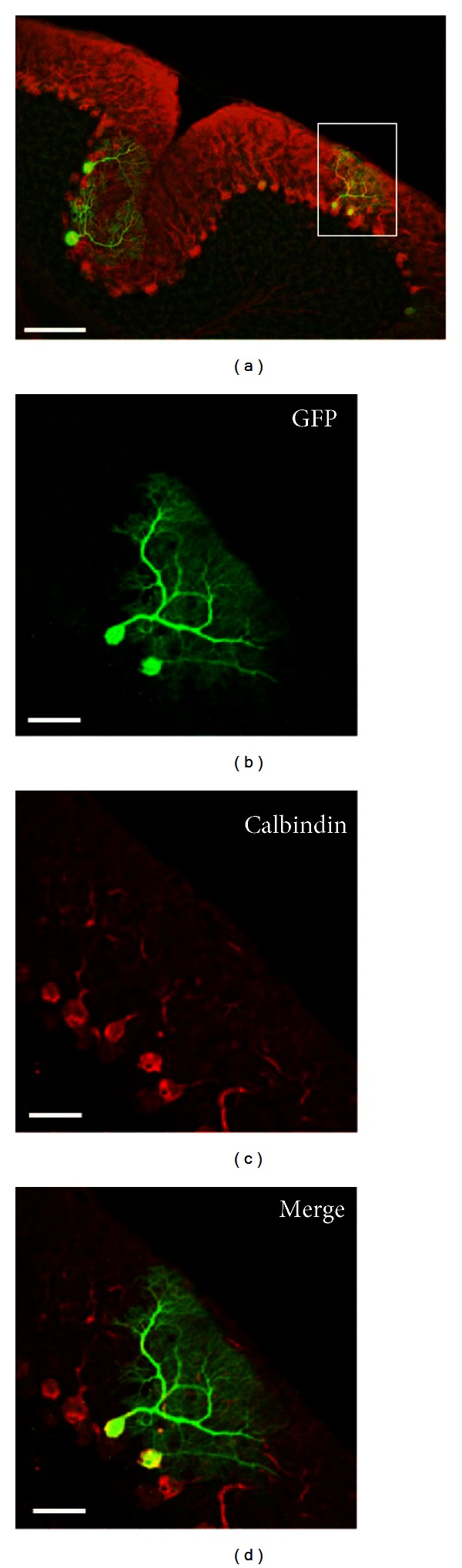
Transduction of cerebellar Purkinje cells. Immunohistochemical detection of GFP and calbindin, a Purkinje cell marker, in mouse cerebellums following intracardiac injections of tyrosine-mutant AAV9/3 vectors that expressed GFP under control of the L7 promoter. (a) An axial section demonstrating colabeling of GFP (green) and calbindin (red) in the cerebellar cortex. ((b)–(d)) High magnification images of the rectangular area outlined in (a). (b) GFP-positive cells (green); (c) Calbindin-positive cells (red); and (d) the merged image of ((b) and (c)). Scale bars: (a) 100 *μ*m; ((b)–(d)) 30 *μ*m.

To investigate if neuron-specific expression could also be obtained with other promoters, we generated yfAAV9/3 vectors where GFP was under control of the cerebellar Purkinje cell-specific L7 promoter [32]. As anticipated, the yfAAV9/3-L7-GFP vectors produced selective GFP expression in Purkinje cells (Figure 5).

### 3.3. Delivery of miRNAs into the Substantia Nigra

**Figure 6:**
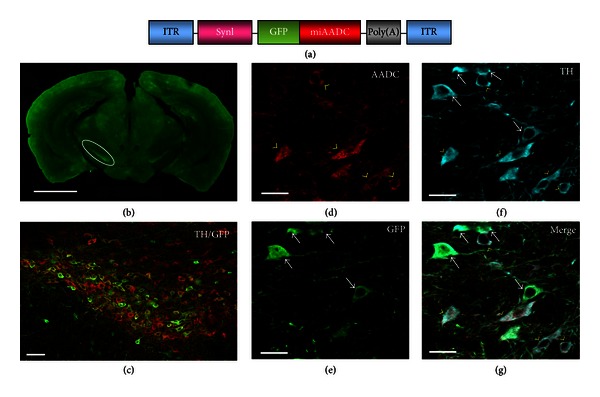
Delivery of miRNAs into the substantia nigra with systemic injections of tyrosine-mutant AAV9/3 vectors. (a) Illustration of the vector construct. The double-stranded pre-miRNA sequence for aromatic l-amino acid decarboxylase (AADC) was placed between the GFP and SV40 poly(A) sequences. ITR: inverted terminal repeat; SynI: synapsin I promoter. (b) A coronal mouse brain section showing a high concentration of GFP-immunoreactive cells in the substantia nigra pars compacta (SNc, circled area). (c) An enlarged image of the SNc subjected to double immunostaining shows colocalization of GFP-positive cells (green) and tyrosine hydroxylase (TH-) positive cells (red). ((d)–(g)) Selective inhibition of AADC expression in SNc neurons. AADC immunoreactivity (red) was not detected in the cells that were positive for both GFP (green) and TH (blue) (arrows). TH-positive cells that were GFP-negative displayed AADC immunoreactivity (arrowheads). Scale bars: (b) 2 mm; (c) 100 *μ*m; ((d)–(g)) 20 *μ*m.

We next asked whether miRNAs could be delivered through systemic injections of the vectors into adult mice. We generated a yfAAV9/3 vector that expressed GFP and the pre-miRNA for AADC under control of the synapsin I promoter (yfAAV9/3-miR-AADC, Figure 6(a)). We administered the yfAAV9/3-miR-AADC vector (8.5 × 10^12^ vectors genome/mouse) to mice with intracardiac injections. Eight weeks after administration, mice were sacrificed, and we examined the expression of AADC, TH, and GFP in neurons of the substantia nigra pars compacta (SNc). Immunohistochemical examinations demonstrated extensive GFP-IR cells in the SNc (Figures 6(b) and 6(c)). AADC immunoreactivity was not detected in cells that expressed both GFP and TH (Figure 6(g), arrows). Furthermore, cells that were TH positive and GFP negative displayed AADC immunoreactivity (Figure 6(g), arrowheads). Taken together, these results suggest that we achieved selective inhibition of AADC expression in the transduced dopaminergic cells.

## 4. Discussion

The BBB is a significant obstacle when attempting to translate gene therapies for diseases that affect broad areas of the CNS. We previously demonstrated that AAV8 vectors that were intravenously administered crossed the BBB and transduced both neurons and glial cells in adult mouse brains. Importantly, these mice had not been pre treated with any osmotic drugs [33]. A sizeable number of studies have focused on finding efficient and safe AAV vectors that can be used to deliver therapeutic genes to large areas of the CNS through systemic administrations. AAV9 vectors, especially double-stranded self-complementary AAV9 (scAAV9) vectors, administered through intravenous means can cross the BBB in mice [13, 16, 18], cats [10], and nonhuman primates [9, 14, 18] and achieve efficient gene delivery in the CNS. The advantage of scAAV9 vectors is that they bypass the need for conversion of the single-stranded AAV genome to double-stranded DNA and thus permit faster and increased expression of the transgene [17]. However, their small packaging capacity compromises the amount of genetic materials that can be inserted into the vectors. The inability to harbour large genes, promoters, and regulatory sequences is a significant disadvantage for many gene delivery applications [34]. Transduction of the CNS was also reported following intrathecal administration of scAAV9 vectors to nonhuman primates [9, 19] and pigs [11]. However, previous studies found that scAAV9 vectors have much greater infectivities of astrocytes than neurons in adult rodents [12, 35] and nonhuman primates [18]. Gene delivery to astrocytes has therapeutic potential for some neurological diseases, such as amyotrophic lateral sclerosis and Parkinson-disease, where astrocytes play active roles in neuronal survival [36, 37]. However, the relatively poor transduction of neurons limits the applications of AAV9 vectors. 

Here, we demonstrated robust transductions of neurons in adult mice by systemic delivery of tyrosine-mutant AAV9/3 vectors with neuron-specific promoters.Surface-exposed tyrosine residues on AAV capsids are critical for infections. Oxidation of tyrosine residues hinders externalisation of the N-terminal portion of the capsid proteins [38]. Mutation of surface-exposed tyrosines to phenylalanines reduces ubiquitination of the capsid proteins and improves the intracellular trafficking to the nucleus [39]. While enhanced gene deliveries to the retinas or spinal cords in mice are achieved with systemic administrations of single tyrosine-mutated AAV2, AAV8, and AAV9 [21, 40, 41], single tyrosine mutations in AAV8 and AAV9 capsids are insufficient for enhanced gene deliveries to the skeletal muscles and hearts [25]. Analyses of AAV2 vectors found that, of several different Y to F mutation combinations, a triple mutant vector (Y444, 500, and 730F) led to the most intense and uniform expression of transgenes in both cultured cells [22] and mice livers [42]. The amino acid residue at position 501 in wildtype AAV9 (residue 500 in AAV2) is phenylalanine, so we used a double tyrosine-mutant form of AAV9(Y446F and Y731F) [26]. Neither mutation led to any sequence changes in the potential assembly activating protein (AAP) gene [43], and the mutant capsids packaged the vectors with titers similar to those of the wild-type capsids. 

Although the CAG promoter achieved extensive transgene expression in the mouse CNS, transduction of antigen-presenting cells with vectors containing ubiquitous promoters may trigger strong cell-mediated immune responses. However, generation of viral vectors with neuron-specific promoters will permit directed expression of therapeutic proteins in diseased neurons.

We targeted SNc neurons to determine whether yfAAV9/3 vectors are capable of functional miRNA delivery. The dopaminergic neurons in the SNc that express both TH and AADC are well defined and well characterised. We found that selective inhibition of AADC was possible with systemic injections of AAV vectors. This approach could provide a novel therapeutic strategy for future studies into Parkinson's disease or basal ganglia functions. 

## 5. Conclusion

We showed that double tyrosine-mutant AAV9/3 vectors significantly enhanced gene delivery to the CNS and that viral-mediated gene expression can be restricted to neurons by incorporating neuron-specific promoters into viral vectors. This approach provides a new methodology for the future development of CNS gene therapies and creation of animal models of neurodegenerative diseases.

## References

[1] Bowers WJ, Breakefield XO, Sena-Esteves M (2011). Genetic therapy for the nervous system. *Human Molecular Genetics*.

[2] High KA, Aubourg P (2011). rAAV human trial experience. *Methods in Molecular Biology*.

[3] Weinberg MS, Samulski RJ, McCown TJ (2013). Adeno-associated virus (AAV) gene therapy for neurological disease. *Neuropharmacology*.

[4] Hadaczek P, Eberling JL, Pivirotto P, Bringas J, Forsayeth J, Bankiewicz KS (2010). Eight years of clinical improvement in MPTP-lesioned primates after gene therapy with AAV2-hAADC. *Molecular Therapy*.

[5] Muramatsu S, Fujimoto K, Kato S (2010). A phase I study of aromatic L-amino acid decarboxylase gene therapy for parkinson’s disease. *Molecular Therapy*.

[6] Bartus RT, Baumann TL, Brown L (2012). Advancing neurotrophic factors as treatments for age-related neurodegenerative diseases: developing and demonstrating, “clinical proof-of-concept” for AAV-neurturin (CERE-120) in Parkinson's disease. *Neurobiology of Aging*.

[7] Christine CW, Starr PA, Larson PS (2009). Safety and tolerability of putaminal AADC gene therapy for Parkinson disease. *Neurology*.

[8] LeWitt PA, Rezai AR, Leehey MA (2011). AAV2-GAD gene therapy for advanced Parkinson’s disease: a double-blind, sham-surgery controlled, randomised trial. *The Lancet Neurology*.

[9] Bevan AK, Duque S, Foust KD (2011). Systemic gene delivery in large species for targeting spinal cord, brain, and peripheral tissues for pediatric disorders. *Molecular Therapy*.

[10] Duque S, Joussemet B, Riviere C (2009). Intravenous administration of self-complementary AAV9 enables transgene delivery to adult motor neurons. *Molecular Therapy*.

[11] Federici T, Taub JS, Baum GR (2012). Robust spinal motor neuron transduction following intrathecal delivery of AAV9 in pigs. *Gene Therapy*.

[12] Foust KD, Nurre E, Montgomery CL, Hernandez A, Chan CM, Kaspar BK (2009). Intravascular AAV9 preferentially targets neonatal neurons and adult astrocytes. *Nature Biotechnology*.

[13] Rahim AA, Wong AM, Hoefer K (2011). Intravenous administration of AAV2/9 to the fetal and neonatal mouse leads to differential targeting of CNS cell types and extensive transduction of the nervous system. *The FASEB Journal*.

[14] Samaranch L, Salegio EA, San Sebastian W (2012). Adeno-associated virus serotype 9 transduction in the central nervous system of nonhuman primates. *Human Gene Therapy*.

[15] Glascock JJ, Osman EY, Wetz MJ (2012). Decreasing disease severity in symptomatic, Smn(−/−);SMN2(+/+), spinal muscular atrophy mice following scAAV9-SMN delivery. *Human Gene Therapy*.

[16] Foust KD, Wang X, McGovern VL (2010). Rescue of the spinal muscular atrophy phenotype in a mouse model by early postnatal delivery of SMN. *Nature Biotechnology*.

[17] McCarty DM (2008). Self-complementary AAV vectors; advances and applications. *Molecular Therapy*.

[18] Gray SJ, Matagne V, Bachaboina L, Yadav S, Ojeda SR, Samulski RJ (2011). Preclinical differences of intravascular AAV9 delivery to neurons and glia: a comparative study of adult mice and nonhuman primates. *Molecular Therapy*.

[19] Ciesielska A, Hadaczek P, Mittermeyer G (2012). Cerebral infusion of AAV9 vector-encoding non-self proteins can elicit cell-mediated immune responses. *Molecular Therapy*.

[20] Delzor A, Dufour N, Petit F (2012). Restricted transgene expression in the brain with cell-type specific neuronal promoters. *Human Gene Therapy Methods*.

[21] Ku CA, Chiodo VA, Boye SL (2011). Gene therapy using self-complementary Y733F capsid mutant AAV2/8 restores vision in a model of early onset Leber congenital amaurosis. *Human Molecular Genetics*.

[22] Li M, Jayandharan GR, Li B (2010). High-efficiency transduction of fibroblasts and mesenchymal stem cells by tyrosine-mutant AAV2 vectors for their potential use in cellular therapy. *Human Gene Therapy*.

[23] Petrs-Silva H, Dinculescu A, Li Q (2011). Novel properties of tyrosine-mutant AAV2 vectors in the mouse retina. *Molecular Therapy*.

[24] Qiao C, Zhang W, Yuan Z (2010). Adeno-associated virus serotype 6 capsid tyrosine-to-phenylalanine mutations improve gene transfer to skeletal muscle. *Human Gene Therapy*.

[25] Cheng B, Ling C, Dai Y (2012). Development of optimized AAV3 serotype vectors: mechanism of high-efficiency transduction of human liver cancer cells. *Gene Therapy*.

[26] Dalkara D, Byrne LC, Lee T (2012). Enhanced gene delivery to the neonatal retina through systemic administration of tyrosine-mutated AAV9. *Gene Therapy*.

[27] Zhang Y, Duan D (2012). Novel mini-dystrophin gene dual adeno-associated virus vectors restore neuronal nitric oxide synthase expression at the sarcolemma. *Human Gene Therapy*.

[28] Dittgen T, Nimmerjahn A, Komai S (2004). Lentivirus-based genetic manipulations of cortical neurons and their optical and electrophysiological monitoring *in vivo*. *Proceedings of the National Academy of Sciences of the United States of America*.

[29] Meyuhas O, Klein A (1990). The mouse ribosomal protein L7 gene. Its primary structure and functional analysis of the promoter region. *Journal of Biological Chemistry*.

[30] Gao G, Vandenberghe LH, Alvira MR (2004). Clades of adeno-associated viruses are widely disseminated in human tissues. *Journal of Virology*.

[31] Li XG, Okada T, Kodera M (2006). Viral-mediated temporally controlled dopamine production in a rat model of Parkinson disease. *Molecular Therapy*.

[32] Wagner W, McCroskery S, Hammer JA (2011). An efficient method for the long-term and specific expression of exogenous cDNAs in cultured Purkinje neurons. *Journal of Neuroscience Methods*.

[33] Nakai H, Fuess S, Storm TA, Muramatsu SI, Nara Y, Kay MA (2005). Unrestricted hepatocyte transduction with adeno-associated virus serotype 8 vectors in mice. *Journal of Virology*.

[34] Wang Y, Ling C, Song L (2012). Limitations of encapsidation of recombinant self-complementary adeno-associated viral genomes in different serotype capsids and their quantitation. *Human Gene Therapy Methods*.

[35] Zhang H, Yang B, Mu X (2011). Several rAAV vectors efficiently cross the blood-brain barrier and transduce neurons and astrocytes in the neonatal mouse central nervous system. *Molecular Therapy*.

[36] Philips T, Robberecht W (2011). Neuroinflammation in amyotrophic lateral sclerosis: role of glial activation in motor neuron disease. *The Lancet Neurology*.

[37] Rappold PM, Tieu K (2010). Astrocytes and therapeutics for Parkinson’s disease. *Neurotherapeutics*.

[38] Horowitz ED, Finn MG, Asokan A (2012). Tyrosine cross-linking reveals interfacial dynamics in adeno-associated viral capsids during infection. *ACS Chemical Biology*.

[39] Zhong L, Li B, Mah CS (2008). Next generation of adeno-associated virus 2 vectors: point mutations in tyrosines lead to high-efficiency transduction at lower doses. *Proceedings of the National Academy of Sciences of the United States of America*.

[40] Petrs-Silva H, Dinculescu A, Li Q (2009). High-efficiency transduction of the mouse retina by tyrosine-mutant AAV serotype vectors. *Molecular Therapy*.

[41] Miyazaki Y, Adachi H, Katsuno M (2012). Viral delivery of miR-196a ameliorates the SBMA phenotype via the silencing of CELF2. *Nature Medicine*.

[42] Markusic DM, Herzog RW, Aslanidi GV (2010). High-efficiency transduction and correction of murine hemophilia B using AAV2 vectors devoid of multiple surface-exposed tyrosines. *Molecular Therapy*.

[43] Naumer M, Sonntag F, Schmidt K (2012). Properties of the adeno-associated virus assembly activating protein. *Journal of Virology*.

